# Role of Serum Amyloid A as a Biomarker for Predicting the Severity and Prognosis of COVID-19

**DOI:** 10.1155/2022/6336556

**Published:** 2022-11-24

**Authors:** Amal A. Abbas, Asma Alghamdi, Sonia Mezghani, Mourad Ben Ayed, Ahmed M. Alamori, Ghazi A. Alghamdi, Wail Bajhmom, Hanan Wajeeh, Salma S. Almutairi, Wafaa M. Radwan

**Affiliations:** ^1^Laboratory Department, King Fahd General Hospital, MOH, Jeddah, Saudi Arabia; ^2^Clinical Pathology Department, Faculty of Medicine, Ain Shams University, Egypt; ^3^Division of Pulmonology, Allergy, and Immunology, Department of Medicine, King Fahd General Hospital, MOH, Jeddah, Saudi Arabia; ^4^Pulmonology, Allergy, and Immunology Department, University of Medicine of Sousse, Tunisia; ^5^Clinical Pathology Department, University of Medicine of Sfax, Tunisia; ^6^Internal Medicine Department of King Fahd General Hospital, MOH, Jeddah, Saudi Arabia; ^7^Clinical Pathology Department, Faculty of Medicine, Menoufia University, Egypt

## Abstract

**Objective:**

To detect biomarkers that can be used to predict COVID-19 severity to identify patients with high probability of disease progression and poor prognosis.

**Methods:**

Of the 102 patients with confirmed COVID-19 who were admitted to King Fahd General Hospital, Jeddah City, Saudi Arabia, from July 1, 2021 to August 5, 2021, 50 were included in this cross-sectional study to investigate the influence of serum amyloid A (SAA) on disease severity and survival outcomes of COVID-19 patients. Dynamic shifts in SAA, C-reactive protein (CRP), white blood cell (WBC), lymphocytes, neutrophils, biochemical markers, and disease progression were examined. At admission, and at three, five, and seven days after treatment, at least four data samples were collected from all patients, and they underwent clinical status assessments.

**Results:**

Critically ill patients showed higher SAA and CRP levels and WBC and neutrophil counts and significantly lower lymphocyte and eosinophil counts compared to the moderately/severely ill patients, especially with regard to disease progression. Similarly, nonsurvivors had higher SAA levels than survivors. The moderately/severely ill patients and the survivors had significantly higher dynamic changes in SAA compared to the critically ill patients and nonsurvivors, respectively, with differences clearly noticed on the fifth and seventh day of treatment. ROC curve analysis revealed that the combination of SAA and CRP was valuable in evaluating the disease progression and prognosis of COVID-19 patients at different time points; however, a combination of SAA and lymphocyte counts was more sensitive for disease severity prediction on admission. The most sensitive parameters for predicting survival on admission were the combination of SAA/WBC and SAA/neutrophil count.

**Conclusions:**

The study findings indicate that SAA can be used as a sensitive indicator to assess the degree of disease severity and survival outcomes of COVID-19 patients.

## 1. Introduction

The coronavirus disease 2019 (COVID-19) pandemic, caused by severe acute respiratory syndrome coronavirus 2 (SARS-CoV-2), continues to take a toll on public health worldwide [1]. A hyperinflammatory process, known as a “cytokine storm,” is considered the cause of many of the fatal illnesses associated with SARS-CoV-2 [2]. These can include acute respiratory distress syndrome (ARDS) and multiple organ failure that lead to COVID-19 aggravation or fatality [3]. Therefore, early diagnosis of COVID-19 is important, as is finding biomarkers that can predict its severity and recovery [4]. Consequently, the search for efficient biomarkers for predicting disease progression from mild to severe in a timely manner is a major area of interest among frontline clinicians [1].

Specific inflammatory indicators have some accuracy in predicting disease severity and mortality [1]. The existing body of research on COVID-19 has demonstrated the use of inflammatory markers, including serum amyloid A (SAA), C-reactive protein (CRP), white blood cell (WBC) counts, lymphocyte and platelet counts, and erythrocyte sedimentation rates as inflammatory indicators [5–7]. SAA is an acute-phase protein that is primarily produced by the liver as a result of proinflammatory cytokine production, mainly IL-1, IL-6, and TNF-*α* which are secreted by activated monocytes [3]. SAA is elevated in all COVID-19 patients, and the mean SAA values are higher in patients who are severely ill than in those who are mildly ill [8]. Shi et al. [9] demonstrated elevated SAA levels in more than 20% of patients, even though their CRP levels were normal and some of the patients had severe pneumonia, indicating a higher sensitivity for SAA in determining the severity of COVID-19. This potential link between disease severity and SAA level was verified by Wang et al. [10] as more significant than that between CRP and ESR. Interestingly, the predictive value for disease progression was higher for the initial SAA level than for the initial CT scan [11].

One hypothesis to explain these findings is that an increase in the SAA level in COVID-19 patients potentially aggravates their clinical state by inducing coagulopathy, decreases in pulmonary and tissue gas exchange, and atherogenesis [12]. A retrospective study of COVID-19 death cases revealed that, among the included serum biomarkers, only SAA was significantly elevated in all patients with fatal outcomes [11], suggesting a role for SAA in COVID-19 pathogenesis and immune-mediated damage progression [13]. An increasing body of evidence therefore suggests that SAA may be useful as an indicator for monitoring disease progression and severity in COVID-19 patients [14, 15].

The aim of the present study was to conduct a systematic investigation of the dynamic changes in SAA and other inflammatory markers in 50 COVID-19 patients. A second goal was to explore the clinical prognostic value of SAA, particularly as a predictor of disease severity and fatality when measured on admission and at different time points after hospitalisation, as these measurements could significantly impact the future management of COVID-19 patients and potential therapeutic strategies. The measurements of SAA levels and other laboratory tests were first conducted in the two groups of patients diagnosed with COVID-19 and classified as moderately/severely and critically ill and then in two emerging groups: survivors and nonsurvivors. The levels of SAA and other inflammatory indicators were monitored in all patients on admission and at different time points after hospital admission, and the dynamic changes were analysed.

## 2. Materials and Methods

### 2.1. Subjects

Prior to the initiation of the study, we obtained ethical approval from the local Institutional Review Board. We conducted a cross-sectional study to investigate the influence of SAA on COVID-19 severity and prognosis in patients hospitalised in our facility (King Fahd General Hospital, Jeddah City, Kingdom of Saudi Arabia (KSA)) from July 1 to August 5, 2021. Patients with suspected COVID-19 symptoms (e.g., cough, fever, dyspnoea, and/or anosmia) and those whose imaging test (e.g. chest X-ray and/or computed tomography) results suggested viral pneumonia were subjected to a real-time polymerase chain reaction test using a nasopharyngeal swab and conducted at the MoH Regional Laboratory, Jeddah, KSA. All the participants were positive for COVID-19 and were hospitalised. In total, samples from 50 individuals were used: 24 patients with moderate to severe symptoms and admitted to the ward section and 26 patients with critically severe symptoms and admitted to the intensive care unit (ICU).

#### 2.1.1. Clinical Condition Assessment Criteria

The COVID-19 Diagnosis and Treatment Plan issued by the Saudi MoH Protocol for Patients Confirmed with COVID-19 [16] lists three types of clinical conditions: mild to moderate, severe, and critically severe.

*(1) Mild to Moderate*. No O_2_ requirement/no evidence of pneumonia but showing other COVID-19 symptoms (e.g., fever).

*(2) Severe*. Showing clinical symptoms of pneumonia (e.g., fever, cough, dyspnoea, and fast breathing) and one of the following:

- Respiratory rate greater than 30/min (adults)
- Blood oxygen saturation less than 90% in room air
- Severe respiratory distress

*(3) Critically Severe*. Showing any of the following symptoms:

- ARDS
- Respiratory failure and requiring ventilation
- Sepsis
- Septic shock

#### 2.1.2. Chest X-Ray Classification

The imaging was divided into four categories: normal/abnormal at admission, deteriorated, improved, and stable upon follow-up.

The patients were treated based on the Saudi MoH Protocol guidelines [16], which include standard treatments, such as early effective oxygen therapy, glucocorticoids, and antiviral and nutritional assistance.

### 2.2. Laboratory Investigations

#### 2.2.1. Sample Collection

Complete blood counts and serum biochemistry tests were conducted at the time of admission. On the first day (before therapy) and on the third, fifth, and seventh day following treatment, 5 mL of fasting venous blood was taken in the early morning for measurement of the levels of inflammatory markers. Serum was separated from the blood at room temperature using high-speed centrifugation and kept at −20°C for testing.

#### 2.2.2. Measurement of SAA and hsCRP

The serum levels of SAA (reference range: <6.4 mg/L) and hsCRP (reference range: 0.0–3.1 mg/L) were measured via immunoturbidimetry in a Siemens Atellica NEPH 630 nephelometer using the Siemens N Latex SAA and N Latex hsCRP assays, respectively (Siemens Healthcare GmbH, Germany).

#### 2.2.3. Measurement of Other Inflammatory Indicators

Serum levels of ferritin (reference range: 20–250 ng/mL) and all the biochemical parameters were measured in an Atellica CH 930 (Siemens Healthineers GmbH, Germany). The plasma fibrinogen concentration (reference range: 1.8 to 3.5 g/L), plasma D-dimer level (reference range: 0.17–0.550 *μ*g/mL), and prothrombin time and activated partial thromboplastin time were measured with a BCS® XP System and Sysmex® CS-5100 Haemostasis System (Siemens Healthineers GmbH, Germany).

### 2.3. Statistical Analyses

The data were analysed using IBM SPSS Statistics (v26.0, IBM Corp., USA, 2019). The quantitative parametric measurements were expressed as mean ± SD, quantitative nonparametric as median and percentiles and categorised data as both numbers and percentages. The tests were conducted using the following procedure: (1) using Student's *t*-test, two independent mean groups were compared for parametric data. The actual difference was reflected by the degree of change owing to a follow-up study (delta change [dC]). Changes occurring throughout the follow-up study were calculated for each patient. The mean dC, calculated by: dC = (Post − Pre)/Pre, was then compared to other groups or correlated with other variables. (2) The Wilcoxon rank-sum test was used to compare the nonparametric data between two independent groups. (3) The ranked Spearman correlation test was used to analyse the relationship between each two variables among each group for nonparametric data. (4) The Chi-squared test was used to investigate the relationship between two variables or to compare two independent groups in terms of categorised data. The error probability was considered significant at 0.05, whereas it was highly significant at 0.01 and 0.001. (5) A receiver operating characteristic (ROC) curve analysis was used to determine the sensitivity and specificity of various biochemical tests in the survivors and nonsurvivors, as well as in patients with moderate/severe and critically severe COVID-19 symptoms. The area under the ROC curve (AUC) was also calculated to evaluate the most distinguishing indicators among the compared groups. (6) A panel (independent parameters) to predict the target parameter (dependent variable) was found using logistic multiregression analysis.

## 3. Results

### 3.1. Patient Characteristics and Clinical Features on Admission

From July 1, 2021 to August 5, 2021, 102 COVID-19 patients were treated at King Fahd General Hospital in Jeddah City. Of those, 50 met the study criteria. The patients' ages ranged from 33 to 92 years, with a mean age of 58 years; 32 were males (64%) and 18 were females (36%). At the time of admission, 24 patients had moderate to severe symptoms (48%) and were admitted to the ward section, whereas 26 patients had critically severe symptoms (52%) and were admitted to the ICU. Based on the clinical progression and survival outcomes, the patients were further stratified into two categories: survivors (38 cases) and nonsurvivors (12 cases).

Consistent with the previous studies [17, 18], comparison between groups in the current investigation regarding patient general characteristics (age, gender, clinical manifestation, and X-ray findings) revealed that the nonsurvivor group was significantly older than the survivor group (*P* < 0.001), and chest X-ray showed a more significant deterioration during follow-up in the critically severe and nonsurvivor groups than in the moderate/severe and survivor groups, respectively. The following were the most common symptoms of the disease onset: shortness of breath (80.0%), fever (42.9%), cough (42.9%), dizziness and headache (24.5%), diarrhoea and vomiting (20%), muscle soreness (16.3%), chest tightness (14.3%), and fatigue (10.2%); other clinical manifestations were not significant (Table 1).

### 3.2. Laboratory Findings of COVID-19 Patients

We further explored the association of inflammatory parameters with severity and mortality in COVID-19 patients by assessing the baseline laboratory findings. The levels of inflammatory indicators, including CRP, SAA, erythrocyte sedimentation rate (ESR), ferritin, and fibrinogen, significantly exceeded the upper limits of the normal ranges in both the moderately/severely and critically ill groups, although no significant difference was found between the two groups. By contrast, other inflammatory indicators, including the leukocyte count, neutrophil count, and D-dimer levels, were significantly higher in the critically severe patients than in the moderate/severe group (*P* < 0.05) (Table 2). However, with the progression of the disease severity, the levels of SAA and CRP, in addition to other inflammatory indices, were significantly higher in the critical group than in the moderate/severe group, as well as in the nonsurvivors compared to the survivors (Supplementary Table 1). Taken together, these results reflected a more profound association between the inflammatory response and expression of acute-phase proteins and both severity and mortality.

Several studies have determined a correlation between the disease severity and lymphopaenia, with severe cases almost always showing a striking decline in lymphocyte numbers [17–19]. Although we observed lymphopaenia in both the moderately/severely and critically ill groups, the difference did not seem significant until the seventh day of hospitalisation. Conversely, from the third to the seventh day of hospitalisation, a drastic lymphopaenia was significantly more evident in nonsurvivors than in survivors (Supplementary Table 1). Eosinopenia was also evident in the moderately/severely and critically ill groups, although it was significantly more severe in the critically ill group and in the nonsurvivors (*P* < 0.05). Eosinopenia is known to manifest as lymphopaenia in COVID-19 pneumonia, although how it contributes to the disease is not yet known. However, the presence of a low percentage of eosinophils could still be used as a biomarker for COVID-19 pneumonia [20]. The pathophysiological role of eosinopenia in COVID-19 still requires further study. Overall, persistently elevated levels of certain inflammatory indicators, mainly SAA and CRP, together with lymphopaenia and eosinopenia, have been closely linked to disease severity and poor outcomes.

By contrast, biochemical parameters, including blood urea nitrogen (BUN), creatinine, uric acid, creatine kinase, creatinine kinase-MB (CK-MB), lactate dehydrogenase (LDH), and blood glucose, were significantly higher (*P* < 0.05) in the critically ill and nonsurvivor groups than in the other groups, possibly due to associated comorbidities, such as uncontrolled diabetes and cardiovascular diseases. Contrarily, the current investigation demonstrated that comorbidities had no impact on the baseline plasma levels of SAA, regardless of the disease condition (Supplementary Table 2).

### 3.3. Dynamic Variations in SAA and CRP in COVID-19 Patients

**Figure 1:**
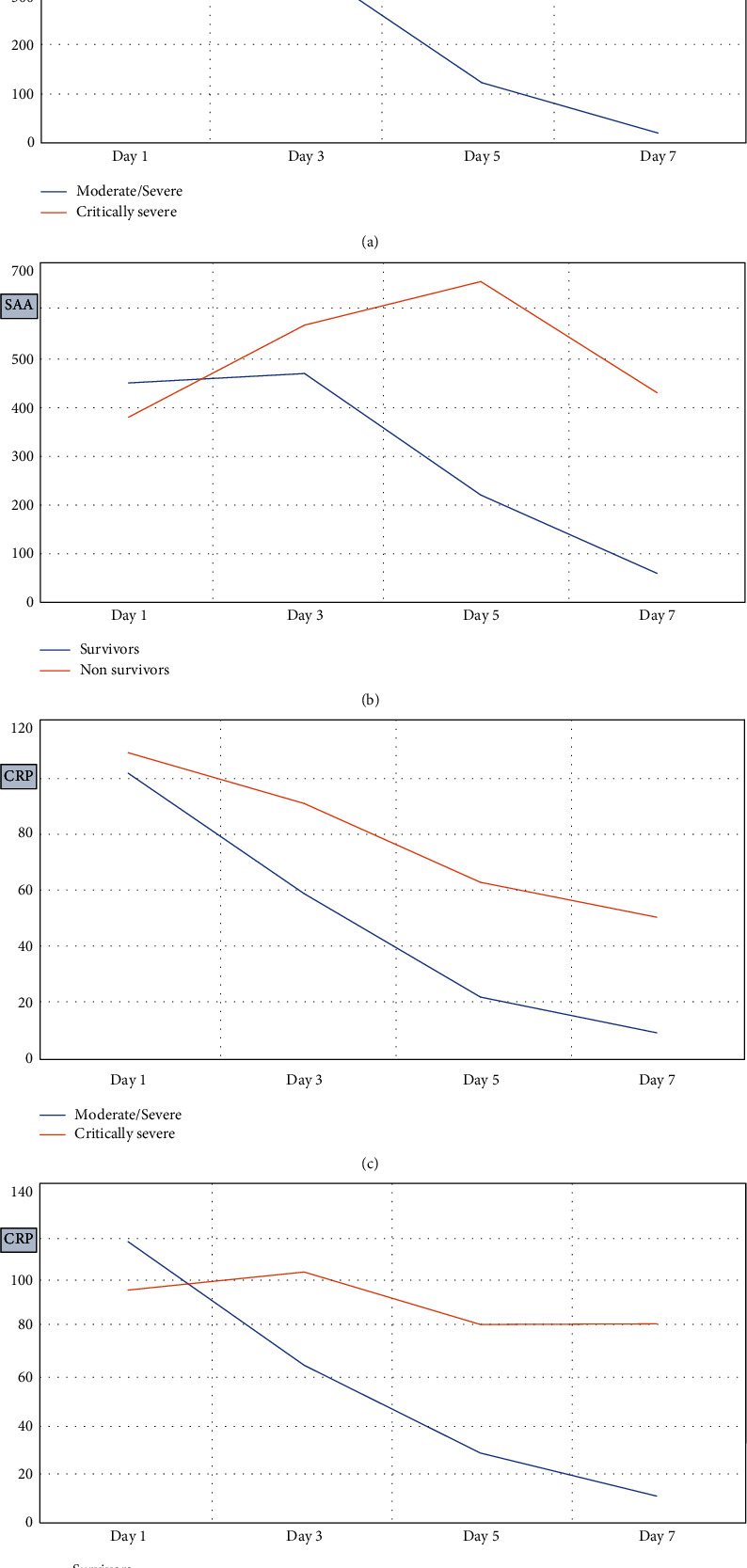
Comparison of dynamic changes of SAA (a, b) and CRP (c, d) levels between moderate/severe and critically severe groups and between survivors and nonsurvivors during hospitalisation.

Laboratories and clinicians are presently directed to pay more attention to dynamic changes in inflammatory markers [18]. Therefore, we studied the dynamic changes in CRP and SAA, as these reflected the change in the patients' conditions over 7 days of hospitalisation (Figure 1). Their health status was more likely to improve when their SAA and CRP levels dropped; therefore, the SAA dynamic changes were compared between the critically ill patients and the moderately/severely ill patients, as well as between nonsurvivors and survivors. Significant and clearly noticeable differences were evident on the fifth and seventh day of treatment (*P* < 0.05). The CRP dynamic changes were significant mainly on the seventh day (Supplementary Table 3). These findings suggest that SAA was a better choice than CRP for predicting the prognosis of patients with COVID-19. We concluded that the dynamic changes in SAA and CRP throughout hospitalisation were consistent with the patients' clinical conditions; hence, these measurements could serve as a new benchmark for monitoring patient prognosis.

### 3.4. Predictive Efficacy of SAA and Other Inflammatory Indicators in the Disease Progression and Survival Outcomes of COVID-19 Patients

**Figure 2:**
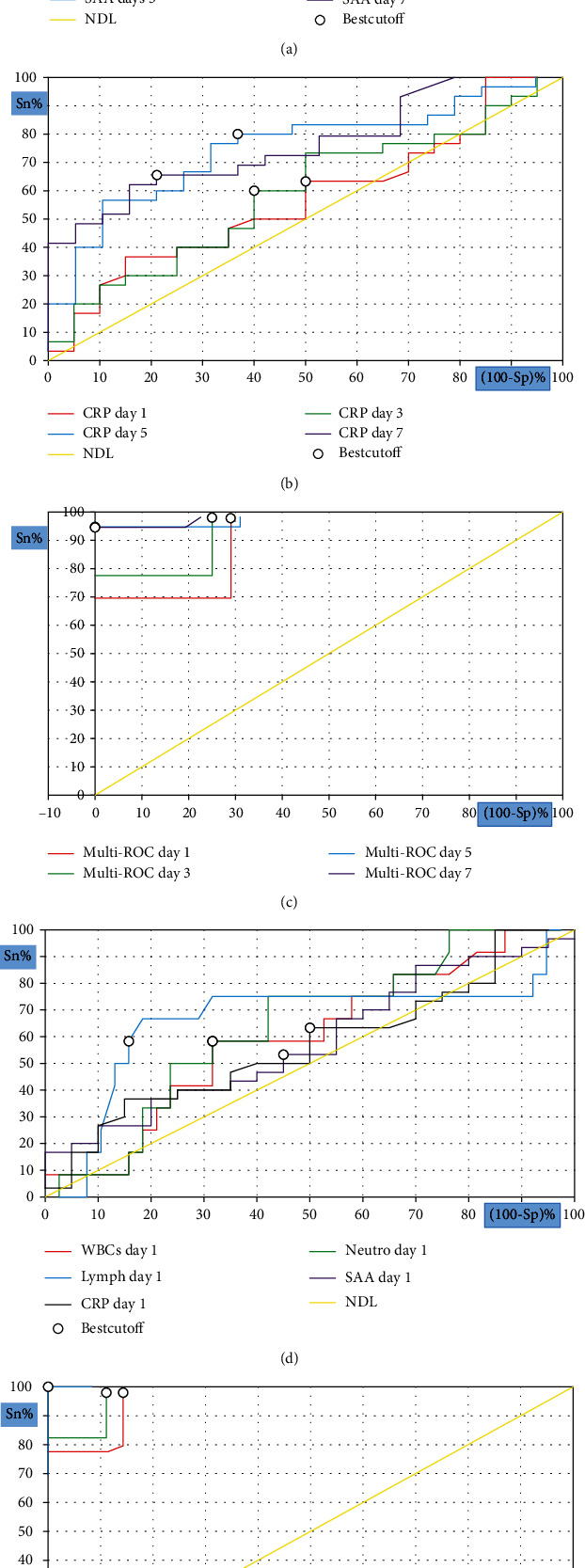
ROC curve analysis for inflammatory indicators to predict disease severity; SAA at different measurement time (a), CRP at different measurement time (b), combined SAA and CRP at different measurement time (c), different biomarkers on admission (d), and combined SAA and different biomarkers on admission (e).

**Figure 3:**
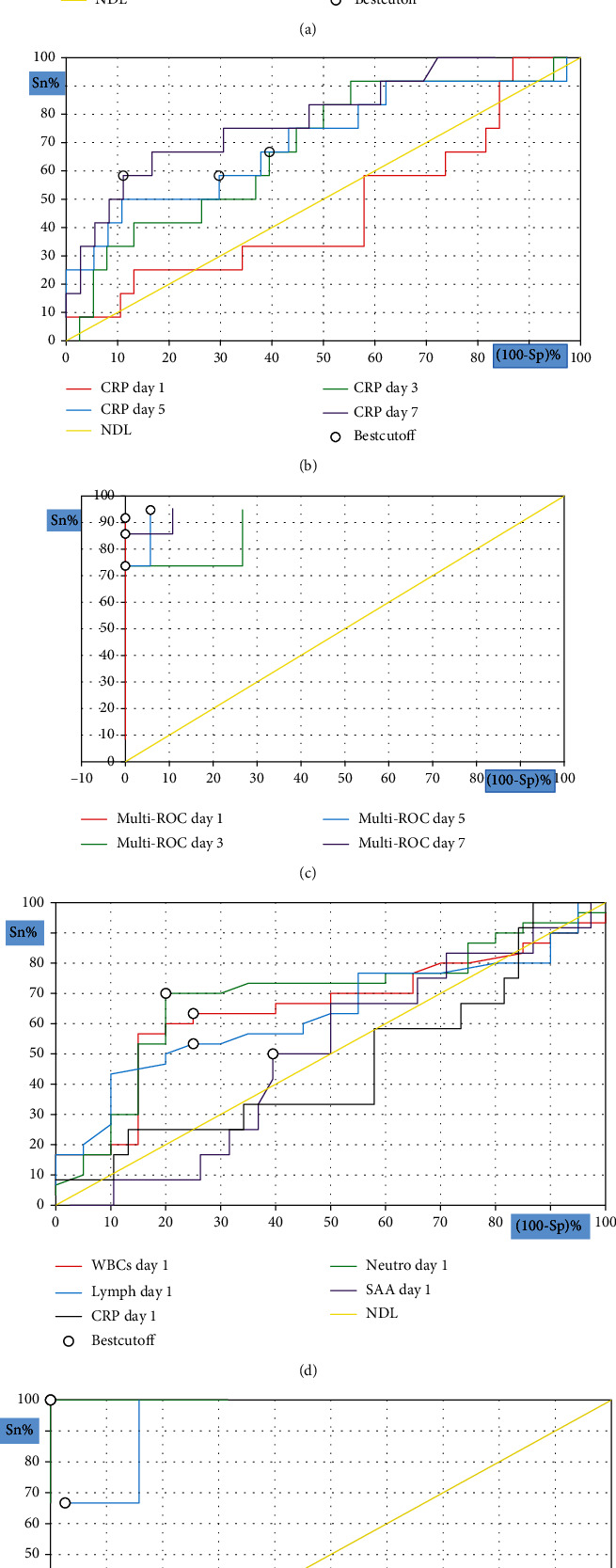
ROC curve analysis for inflammatory indicators to predict mortality; SAA at different measurement time (a), CRP at different measurement time (b), combined SAA and CRP at different measurement time (c), different biomarkers on admission (d), and combined SAA and different biomarkers on admission (e).

The ROC curve was then utilised to determine the early-warning efficiency of SAA and other inflammatory markers and their highest predictive value for COVID-19 disease progression and survival. The analysis results of the ROC curve were used to calculate the AUCs and cut-off values according to their specificity and sensitivity as predictive factors (Table 3, Figures 2 and 3). A study conducted by Mo et al. [21] previously used logistic regression analysis to demonstrate that SAA can be used as an independent and accurate predictor of COVID-19 outcome. However, we must clarify that we found the combination of SAA and CRP was more sensitive for predicting disease progression risk at various time periods. Nevertheless, the combination of lymphocyte count and SAA was by far the most sensitive parameter for predicting the severity on admission. When the cut-off value of the lymphocyte count was 0.76 × 10^9^/L, the cut-off value for the SAA level was 150 mg/L, with a sensitivity and specificity of 100%. The most predictive parameters for determining survival on admission were the combination of SAA and WBC count or the combination of SAA and neutrophil count. These combinations had the highest sensitivity and specificity (100%) and an SAA cut-off level of 152 mg/L when the WBC and neutrophil counts were 7.97 × 10^9^ and 6.32 × 10^9^/L, respectively. Taken together, the use of the combination of SAA and CRP levels, along with WBC, lymphocyte, and neutrophil counts, greatly increased the sensitivity and specificity of disease severity and mortality prediction for COVID-19 in the present study.

## 4. Discussion

The COVID-19 outbreak has had a global impact, and the disease has a wide spectrum of clinical manifestations ranging from mild to life threatening. A majority of COVID-19 patients are asymptomatic or have mild flu-like symptoms, but one-fifth of the cases are severe or critically severe [22], and the probability of poor outcomes increases dramatically as patients proceed to a more severe or critical stage [23]. Approximately 10–15% of mild COVID-19 patients progress to the severe stage, and 15–20% of the severe cases proceed to the critical stage, with many of those in the critical group requiring hospitalisation (i.e., ICU) [24]. Furthermore, critical COVID-19 has a significant link to mortality [25]. Therefore, preventing the progression of the disease from mild or moderate to more severe stages could represent a feasible approach for reducing disease mortality [4]. Thus, the identification of biomarkers that can accurately predict the likelihood of disease progression in COVID-19 patients is critical.

In this study, we investigated the findings of laboratory tests and inflammation-related biomarkers in COVID-19 patients at the time of hospitalisation and again at three, five, and seven days after therapy. On admission, we found that only the WBC count, neutrophil count, and D-dimer were significantly higher in the critically ill group than in the moderately/severely ill group, whereas other inflammatory indicators, mainly CRP and SAA, became significantly different among the studied groups with the progression of disease severity. In line with our findings, several investigations revealed that COVID-19 cases in the ICU had considerably higher numbers of white blood cells and neutrophils, in addition to elevated levels of CRP and other inflammatory markers when compared with the non-ICU cases [26, 27]. Other studies have reported that severely ill patients with COVID-19 tended to have greater levels of proinflammatory cytokines, particularly interleukin- (IL-) 6 [7, 28–30]. Cases with poor prognosis were also characterised by high levels of cytokines [30–32]. These cytokines are secreted mainly by dendritic cells (DCs) and macrophages, which induce the infiltration and recruitment of proinflammatory Th17 cells. Acute respiratory distress syndrome (ARDS) and T-cell overactivation were discovered in the lungs of a COVID-19 deceased. This phenomenon is brought on by an increase in T-helper (Th) 17 cells and strong CD8+ T-cell cytotoxicity [33]. The innate and adaptive immunological responses triggered by SARS-CoV-2 infection result in uncontrolled inflammatory reactions, which in turn generate a cytokine storm [34] that can result in vascular leakage, epithelial and endothelial cell apoptosis, diffuse alveolar damage, ARDS, and even death [35].

Especially in patients with severe illness, COVID-19 is likely to cause a decrease in major T lymphocyte subsets [7, 29, 30]. However, several research studies have presented controversial findings [7, 36]. We observed significant lymphopaenia only in patients with critical illness and in nonsurvivors, especially in conjunction with disease progression. In these patients, several factors may contribute to this picture, such as the ability of SARS-CoV-2 to trigger the P53 signalling pathway and lymphocyte death [37] and to selectively induce macrophages to produce IL-6. The latter response directly promotes lymphocyte necrosis [38] and infection of macrophages in the spleens and lymph nodes, thereby causing splenic nodule atrophy and lymph follicle depletion by promoting activation-induced cell death through Fas/FasL interactions [39]. In addition, increased lymphocyte expression of programmed death inhibitory receptor 1, together with its ligand, blocks signalling pathways and causes the inhibition of lymphocyte proliferation and differentiation [40].

The eosinophil count was significantly lower in the critically ill group and nonsurvivors at admission and during follow-up. Based on specific studies, the consumption of eosinophils induced by SARS-CoV-2 is linked to CD8 T-cell depletion. A decrease in circulating eosinophils was associated with acute infection, and some eosinophil granule proteins, such as eosinophil-cationic protein, eosinophil-peroxidase, and eosinophil-derived neurotoxin, had antiviral properties. In individuals with severe illness and a poor prognosis, eosinopenia may potentially be linked to a high viral load [41]. These findings may offer some explanation regarding the cause of eosinopenia in the affected groups of the current investigation.

The clinical value of SAA, a marker of inflammation, has been attracting increasing attention during the COVID-19 pandemic. An increasing number of studies have demonstrated the clinical value of SAA over many other biomarkers in predicting and monitoring disease progression [1, 4, 5, 42]. SAA may contribute to the pathogenesis of COVID-19 by binding to fibrinogen, resulting in atypical coagulopathy [43]. It also binds to apoB-containing lipoproteins, causing changes in the composition and functionality of high-density lipoproteins [44] and red blood cell agglutination. In combination, these changes contribute to embolic and multiple infarction events in COVID-19 patients [45]. SAA can also enhance inflammatory reactions, albeit at low concentrations, by stimulating chemokine activation and triggering chemotaxis [46, 47]. Hence, it can be used as a prognostic marker for tissue damage or acute infections [48–50] and to track the progression of respiratory illnesses [51, 52].

Several proinflammatory disorders, such as liver disease, autoimmune disease, diabetes, obesity, atherosclerotic cardiovascular disease, and cancer, have been linked to an increase in SAA concentrations [53]. The current study, however, showed that comorbidities, regardless of disease status, had no effect on the baseline plasma level of SAA. Similarly, a retrospective investigation of COVID-19 in acute and convalescent individuals found no change in the plasma level of SAA between patients with and without comorbidities, regardless of the severity of the condition. This suggests that the inflammatory response brought on by SARS-CoV-2 is only minimally affected by underlying diseases [54].

Although we reported that the baseline levels of SAA and CRP were not significantly different between the critically ill and moderately/severely ill groups or between survivors and nonsurvivors, we observed dynamic changes in SAA levels during the 3–7 days of hospitalisation, demonstrating that levels of SAA significantly decreased in the survivor and moderately/severely ill groups, whereas the nonsurvivor and critically ill groups continued to have high levels, particularly at days 5 and 7. This pattern could imply that patients with successively decreasing SAA levels had a better prognosis than those with consistently higher levels, indicating a statistically significant relationship between dynamic changes in SAA and prognosis. A similar trend has been validated by Li et al., who reported that patients with consistently trending downward SAA and CRP levels were more likely to have improved conditions than were those with continuously rising SAA levels [18]. This phenomenon could be attributed to the activation of the body's inflammatory response, which increases the ability of hepatocytes to produce large amounts of SAA [55, 56].

Recent scientific discoveries have revealed that severely/critically ill COVID-19 patients have high blood levels of interleukin-1*β*, interferon-*γ*, interferon gamma-induced protein10, chemotactic protein-1, macrophage inflammation protein-1, tumour necrosis factor–*α*, and other cytokines, which stimulate the production of SAA [28, 35]. An association has been reported between elevated SAA levels and mortality in COVID-19, and nonsurvivors with increased SAA levels demonstrated accelerated immune-mediated damage [28]. Notably, in the present study, dynamic CRP changes became significant only on the seventh day. According to these findings, SAA was a better predictor of COVID-19 patient outcome than CRP. This assumption was supported further by a systematic review and meta-analysis [53] that looked at the magnitude and rate of increase in SAA concentrations during the acute-phase response and reported a higher level of SAA than other inflammatory indicators, particularly CRP. Furthermore, due to the shorter half-life of SAA than CRP, concentrations of SAA typically recover to baseline levels more quickly. These features point to a distinct pathophysiological function for SAA that might complement data provided by other biomarkers, such as CRP, in clinical settings.

The ROC curve was used to assess the predictive power of CRP, SAA, and WBC and lymphocyte and neutrophil counts in severe COVID-19 patients and their survival outcomes. We must clarify that the combination of SAA and CRP was more sensitive for predicting the risk of disease progression and survival at different time points. The combined predictive probabilities of SAA and CRP levels on admission for disease severity and survival outcomes were 0.852 and 0.925, respectively. Interestingly, the combined predictive probability of SAA levels and lymphocyte counts was the most sensitive indicator for predicting the risk of disease severity at the first time point, as it had the highest AUC (1.00). Similarly, the combined predictive probabilities of SAA and WBC counts and SAA and neutrophil counts had the highest AUC for survival at admission. These results indicate that the use of combined predictive factors has a high prognosis value. In addition, the cut-off points of SAA and other biomarkers can be used to manage the preliminary warning signs of severe COVID-19 and improve survival outcomes. These findings have been confirmed by recent studies that have evaluated the clinical value of SAA as a reliable indicator for distinguishing critically severe from moderate cases and survivors from nonsurvivors [17, 18, 57].

This study has some limitations. First, this was a single-centred study with a small population. A large sample, including data from multiple centres, should be used to verify the current findings. Second, because of the epidemic's onset, we lacked sufficient data on healthy patients to serve as baseline controls. Therefore, further studies are required to overcome these limitations. However, the main findings of the present work offered modest yet significant new information that may be used to estimate the outcome of COVID-19 through dynamic monitoring of the prognostic indicators, with potential to improve the survival rate of COVID-19.

## 5. Conclusion

Based on the study results, SAA can be used as a sensitive indicator for determining disease severity and prognosis over more commonly used biomarkers in COVID-19 patients. Furthermore, the combined predictive probability of SAA and CRP levels at various time points, in addition to SAA and WBC and neutrophil and lymphocyte counts on admission, can significantly improve the sensitivity and specificity of disease severity prediction. Therefore, monitoring the dynamic changes in SAA levels is important for choosing an effective diagnostic and treatment plan, especially at the early stage of the disease, to improve survival outcomes.

## Figures and Tables

**Table 1:** Clinical characteristics of the studied patient groups upon admission and their follow-up chest X-rays.

Variables	Moderate/severe group (*n* = 24)	Critically ill group (*n* = 26)	*t*/*χ*^2^ value	*P* value	Survivor group (*n* = 38)	Nonsurvivor group (*n* = 12)	*t*/*χ*^2^ value	*P* value
Age (years)	55 (44.3–68.3)	58.5 (46.7–68.5)	-0.262	0.79	54 (41.8–65)	69 (55.8–79.75)	-2.773	**0.006**
Male/female	14/10	18/8	0.643	0.42	23/15	9/3	0.829^a^	0.362
Respiratory rate (bpm)	21.36 ± 2.498	20.56 ± 3.129	0.888	0.38	21.27 ± 3.129	20.2 ± 1.135	1.581	0.122
Systolic pressure (mmHg)	127 ± 12.968	130.25 ± 18.7	-0.654	0.51	128.79 ± 15.091	127.6 ± 18.65	0.184	0.857
Diastolic pressure (mmHg)	74.87 ± 9.251	73.35 ± 11.43	0.475	0.63	74.97 ± 10.646	71.5 ± 8.618	1.053	0.306
SpO_2_ (%)	95.67 ± 1.834	91.2 ± 9.639	2.274	**0.03**	94.76 ± 3.113	89.17 ± 13.21	1.453	0.173
Body temperature (°C)	37.07 ± 0.495	37.11 ± 0.621	-0.203	0.84	37.13 ± 0.589	36.95 ± 0.433	1.042	0.31
*Clinical manifestationn(%)*								
Fever	13 (54.2)	8 (32.0)	2.457^a^	.117	18 (48.6)	3 (25)	2.069^a^	0.150
Cough	15 (62.5)	16 (61.0)	0.411^a^	.624	18 (48.6)	3 (25)	2.069^a^	0.150
Dizziness and headache	7 (29.2)	5 (19.2)	0.556^a^	.456	9 (24.3)	3 (25)	0.002^a^	0.962
Muscle soreness	7 (29.2)	8 (30.7)	5.677^a^	.217	7 (18.9)	1 (8.3)	0.743a	0.389
Shortness of breath	20 (83.3)	20 (76.9)	0.321^a^	.571	30 (78.9)	10 (83.3)	0.110^a^	0.741
Fatigue	3 (12.5)	2 (8.0)	.271^a^	.603	4 (10.8)	1 (8.3)	0.061^a^	0.805
Chest tightness	5 (20.8)	2 (8.0)	1.647^a^	.199	5 (13.5)	2 (16.7)	0.074^a^	0.786
Diarrhoea	5 (20.8)	5 (19.2)	.020^a^	.887	7 (18.9)	3 (25)	0.247^a^	0.619
*Comorbidityn(%)*								
Diabetes	8 (33.3)	13 (52.0)	1.742^a^	.990	14 (37.8%)	7 (58.3%)	1.554^a^	0.213
Hypertension	11 (47.8)	12 (48.0)	.000^a^	.966	16 (44.4%)	7 (58.3%)	.696^a^	0.404
COPD	2 (8.3)	2 (8.0)	.002^a^	.672	3 (8.1%)	1 (8.3%)	.001^a^	0.98
Cerebrovascular disease	2 (8.3)	3 (12)	.180^a^	.672	2 (5.4%)	3 (25.0%)	3.797^a^	**0.05**
*X-ray evaluation and classification*:								
DAY 1 abnormal	18 (75)	23 (88)	2.641^a^	.450	31 (86.1)	10 (83.3)	2.341a	0.505
DAY 3								
Deteriorated	0 (0.0)	9 (60)	7.200^a^	**.027**	4 (50.0)	5 (41.7%)	0.741a	0.690
Improved	1 (20)	0 (0.0)			0 (0.0)	1 (8.3)		
Stable	4 (80)	6 (40)			4 (50.0)	6 (50.0)		
DAY 5								
Deteriorated	0 (0.0)	13 (65)	10.807^a^	**.013**	4 (21.1)	9 (90.0)	12.719a	**0.005**
Improved	2 (22)	1 (5.0)			3 (15.8)	0 (0.0)		
Stable	6 (66.7)	5 (25.0)			10 (52.6)	1 (10.0)		
DAY 7								
Deteriorated	0 (0.0)	14 (66.7)	6.151a	**.046**	5 (33.3)	9 (90.0)	8.135a	**0.017**
Improved	2 (50.0)	3 (14.3)			5 (33.3)	0 (0.0)		
Stable	2 (50.0)	4 (19)			5 (33.3)	1 (10.0)		

**Table 2:** Analysis and comparison of various inflammatory and biochemical markers of all COVID-19 patients at time of admission.

Laboratory parameters	Moderate/severe group (*n* = 24)	Critically ill group (*n* = 26)	*Z*/*t* value	*P* value	Survivor group (*n* = 38)	Nonsurvivor group (*n* = 12)	*Z*/*t* value	*P* value
Leukocyte count (10^9^/L)	5.95 (4.625–7.8025)	8.68 (5.225–11.5125)	-2.088	**0.037**	6.6 (4.675–9.295)	8.455 (5.53–11.12)	-0.966	0.334
Neutrophil count (10^9^/L)	4.38 (3.03–5.6425)	7.3 (3.775–10.05)	-2.525	**0.012**	4.5 (3.16–7.53)	7.365 (4.18–9.25)	-1.408	0.159
Lymphocyte count (10^9^/L)	0.91 (0.715–1.1225)	0.75 (0.6375–0.991)	-1.34	0.18	0.9 (0.7–1.1)	0.665 (0.52–1.1)	-1.659	0.097
Monocyte count (10^9^/L)	0.41 (0.1875–0.6)	0.385 (0.23–0.7975)	-0.165	0.869	0.41 (0.26–0.6325)	0.29 (0.2–0.972)	-0.375	0.708
Eosinophil count (10^9^/L)	0.03 (0.0025–0.0475)	0.0005 (0–0.01)	-2.725	**0.006**	0.02 (0.–0.04)	0 (0–0.01)	-2.379	**0.017**
Platelet count (10^9^/L)	193 (145.25–252.25)	220 (170.5–300.5)	-0.893	0.372	210 (154.25–305)	200 (171.25–255)	-0.239	0.811
SAA (mg/L)	452.5 (105.525–950.5)	411.5 (170.75–970.25)	-0.534	0.593	452.5 (132.75–953.5)	382 (210.75–891)	-0.034	0.973
CRP (mg/L)	104 (72.3–139.5)	107.45 (58.93–171.25)	-0.33	0.741	116 (76.9–150)	96 (57.175–166)	-0.363	0.716
Erythrocyte sedimentation rate (mm/h)	44 (29.75–70.75)	48 (39.5–52)	-0.085	0.932	43 (34.5–68.25)	51 (49–72.5)	-0.753	0.451
Haemoglobin (g/L)	12.588 ± 2.2447	12.481 ± 2.0603	0.175	0.862	12.492 ± 2.1358	12.658 ± 2.1973	0.23	0.821
Prothrombin time (s)	12.3859 ± 1.62268	11.9196 ± 1.23616	1.104	0.276	12.2972 ± 1.46103	11.6417 ± 1.26164	1.496	0.149
Activated partial thromboplastin time (s)	31.5455 ± 4.99082	30.7292 ± 5.82119	0.523	0.603	31.4 ± 5.11189	30.2133 ± 6.40238	0.583	0.568
D-dimer (mg/L)	0.603 (0.48–1.81)	2.175 (1.05–8.8725)	-2.903	**0.004**	1 (0.5–2.5)	4.65 (1.8425–31.5125)	-2.665	**0.008**
Blood urea nitrogen (mg/dL)	14.64 (10.325–18.475)	27.275 (14.403–53.125)	-2.845	**0.004**	15 (11.23–23.73)	37.1 (27.2–75.1)	-3.862	**<0.001**
Creatinine (mg/dL)	0.945 (0.71–1.2275)	1.49 (0.99825–2.065)	-2.913	**0.004**	1.005 (0.7175–1.3625)	1.55 (1.445–1.8625)	-3.022	**0.003**
Uric acid (mg/dL)	4.65 (3.875–6.85)	6.5 (4.6625, 8.05)	-2.142	**0.032**	4.75 (3.9–6.95)	7.35 (5.625–8.15)	-2.37	**0.018**
Albumin (g/L)	3.0754 ± 0.50186	3.2354 ± 0–5241	-1.102	0.276	3.1321	3.2425 ± 0.469	-0.687	0.5
Total protein (g/L)	6.5389 ± 0.64729	6.4753 ± 0.65732	0.297	0.769	6.5362	6.3975	0.505	0.624
Aspartate aminotransferase (U/L)	59.035 (31.5–76.75)	51.95 (38.25–70.5)	-0.114	0.909	59.035 (32-84.7)	51.95 (44–59.25)	-0.393	0.694
Alanine aminotransferase (U/L)	40 (25–66)	36 (21–82.75)	-0.26	0.794	34 (22.565–68)	42.5 (18–92.5)	-0.174	0.862
Total bilirubin	0.5 (0.39–0.675)	0.4765 (0.3–0.7225)	-0.439	0.661	0.5 (0.3275–0.6675)	0.5 (0.325–0.95)	-0.627	0.531
Creatine kinase (U/L)	95 (43–212)	307 (121.25–699)	-2.831	**0.005**	146 (51.5–314)	307 (179.5–804.5)	-2.041	**0.041**
LDH (U/L)	413 (322–554)	489 (409–857)	-1.791	0.073	454 (383.5–566)	448 (363–876)	-0.314	0.753
Glucose (mg/dL)	123.15 (100.75–183.25)	198 (130.5–305)	-2.59	**0.01**	132 (103.75–201)	224 (162–420)	-2.396	**0.017**
CK-MB	0.39 (0.09–0.68)	0.885 (0.415–2.1175)	-2.215	**0.027**	0.41 (0.1425–0.82)	0.95 (0.95–0.95)	-1.023	0.307
Ferritin	936.5 (296.5–1737.5)	439.5 (145.75–686)	-0.961	0.337	439.5 (318–1625.5)	412.5 (145.25–700)	-1.019	0.308
Fibrinogen (g/L)	5.395 (4.26–5.975)	5.4 (4.31–6.4)	-0.135	0.893	5.73 (4.75–6.5)	5 (2.33–6.4)	-1.214	0.225

SAA: serum amyloid A; CRP: C-reactive protein; CK-MB: creatine kinase-MB; LDH: lactate dehydrogenase. Data are expressed as mean ± SD, median (25 to 75% percentile), *P* value < 0.05 is significant.

**Table 3:** Analysis of the effectiveness of SAA and different biomarkers for predicting the severity and mortality of COVID-19 patients.

Days	Markers	Survival prediction						Severity prediction					
		AUC	SE	95% CI	Cut-off value	SP	SN	AUC	SE	95% CI	Cut-off value	SP	SN
1^st^ day	SAA	0.470	0.097	0.280–0.661	347	60.5	50	0.580	0.084	0.416–0.744	411	55	53.3
	CRP	0.439	0.098	0.247–0.630	—	—	—	0.518	0.084	0.352–0.683	95.5	50	63.3
	WBC count	0.648	0.087	0.478–0.817	7.97	68.4	58.3	0.570	0.084	0.406–0.735	6.7	75	63.3
	Neutrophil count	0.688	0.082	0.528–0.849	6.32	68.4	58.3	0.611	0.083	0.448–0.773	4.57	80	70
	Lymphocyte count	0.583	0.092	0.402–0.763	0.68	84.2	58.3	0.634	0.082	0.473–0.794	0.76	75	53.3
3^rd^ day	SAA	0.579	0.092	0.398–0.760	514	57.9	58.3	0.635	0.082	0.475–0.795	467	60	63.3
	CRP	0.656	0.086	0.488–0.824	90	60.5	66.7	0.538	0.084	0.373–0.704	71	60	60
5^th^ day	SAA	0.717	0.078	0.564–0.871	579	89.2	58.3	0.741	0.074	0.596–0.887	124	52.6	90
	CRP	0.680	0.083	0.518–0.843	49.6	70.3	58.3	0.698	0.078	0.545–0.851	25.9	63.2	80
7^th^ day	SAA	0.713	0.079	0.559–0.867	316	80.6	75	0.690	0.079	0.536–0.844	26.9	63.2	89.7
	CRP	0.619	0.089	0.444–0.794	74.7	88.9	58.3	0.550	0.084	0.385–0.715	12.4	78.9	65.5
1^st^ day	SAA+CRP	0.925	0.037	0.853–0.997	347 + 40	100	91.7	0.852	0.074	0.706–0.998	411 + 11.5	71	97.8
	SAA+WBCs	1.000	0.000	1.000–1.000	152 + 7.97	100	100	0.887	0.053	0.784–0.990	16.3 + 6.7	85.7	98
	SAA+neutrophil count	1.000	0.000	1.000–1.000	152 + 6.32	100	100	0.880	0.054	0.774–0.986	16.3 + 4.57	88.9	98
	SAA+lymphocyte count	0.867	0.051	0.766–0.968	16.3 + 0.68	97.4	66.7	1.000	0.000	1.000–1.000	150 + 0.76	100	100
3^rd^ day	SAA & CRP	0.864	0.052	0.762–0.966	514 + 183	100	73.7	0.884	0.067	0.753–1.015	467 + 10.5	75	98
5^th^ day	SAA & CRP	0.884	0.047	0.791–0.977	579 + 174	100	73.7	0.907	0.061	0.788–1.026	124 + 140	100	94.7
7^th^ day	SAA & CRP	0.902	0.043	0.818–0.986	316 + 142	100	85.7	0.920	0.057	0.809–1.031	26.9 + 70.4	100	94.5

SAA: serum amyloid A; CRP: C-reactive protein; WBC: white blood cells; AUC: areas under the ROC curves; SE: standard error; CI: confidence interval.

## Data Availability

The data used to support the findings of this study are included within the article and supplementary information file.

## Supplementary Materials

Supplementary Materials Supplementary Table 1: comparison of inflammatory indicators between moderate/severe and critically severe and survivor and nonsurvivor groups at the third, fifth, and seventh day of hospitalization. Supplementary Table 2: comparison of initial SAA levels between patients with and without comorbidities across different groups. Supplementary Table 3: dynamic changes of SAA and CRP in COVID-19 patients.
