# Supplementary material for: Role of Serum Amyloid A as a Biomarker for Predicting the Severity and Prognosis of COVID-19
Source: J Immunol Res. 2022 Nov 24;2022:6336556. doi: 10.1155/2022/6336556 (PMC9715339; doi:10.1155/2022/6336556)
Supplement: Supplementary Materials — Supplementary Table 1: comparison of inflammatory indicators between moderate/severe and critically severe and survivor and nonsurvivor groups at the third, fifth, and seventh day of hospitalization. Supplementary Table 2: comparison of initial SAA levels between patients with and without comorbidities across different groups. Supplementary Table 3: dynamic changes of SAA and CRP in COVID-19 patients. [file 6336556.f1.docx]

Supplementary Table 1: Comparison of inflammatory indicators between moderate/severe, critically severe & survivors and non-survivors groups at the third, fifth and seventh days of hospitalization.

| Variables | days of treatment | Severe group (*n* = 24) | Critically ill group (*n* = 26) | *Z*/ *t* value | *P* value | survivor | Non survivor | Z/ t value | P value |
| --- | --- | --- | --- | --- | --- | --- | --- | --- | --- |
| Leukocyte count (10^9^/L) | Day 3 | 6.38 (4.57,8.675 ) | 8.59 (5.885 , 12.355 ) | -1.903 | 0.057 | 7.22 (4.59,12.06 ) | 8.3 (6.08 , 10.32 ) | -0.727 | 0.467 |
|  | Day 5 | 7.555 (5.3325 , 10.58) | 10.77 (7.6625 , 4.3125) | -2.621 | 0.009** | 8.24 (5.73 , 12.54) | 10.77 (8.3 , 13.24) | -1.613 | 0.107 |
|  | Day 7 | 7.095(5.1775 ,10.2025) | 11.48 ( 8.48 , 14.635) | -2.93 | 0.003** | 8.13(5.84 ,12.29) | 11.4 ( 8.97 , 12.59) | -1.174 | 0.24 |
| Neutrophil count (10^9^/ L) | Day 3 | 4.575 (2.8225 , 6.7875) | 7.2 (4.525 , 9.635) | -2.32 | 0.02* | 5.25 (2.90 , 7.99) | 6.8 (5.05 , 9.02) | -1.363 | 0.173 |
|  | Day 5 | 5.65 (3.135 , 8.57 ) | 8.865 (6.4625 , 13.025) | -2.816 | 0.005** | 6.41 (3.73 , 9.56 ) | 9.7 (6.55 , 11.96) | -2.01 | 0.044* |
|  | Day 7 | 4.895(3.6 , 7.7275) | 9.87 (6.605 , 12.98 ) | -3.7 | <0.001** | 5.55(4.21 , 10.07) | 9.78 (7.88 , 11.56 ) | -1.95 | 0.051 |
| Lymphocyte count (10^9^/ L) | Day 3 | 1.12(0.7305 , 1.3125 ) | 0.705 (0.4925 , 1.125 ) | -1.781 | 0.075 | 1.1 (0.69 , 1.26 ) | 0.59 (0.40 , 0.84 ) | -2.602 | 0.009** |
|  | Day 5 | 1.07 (0.7275 , 1.5525 ) | 0.82 (0.4975, 1.58 ) | -1.117 | 0.264 | 1.07 (0.705 , 1.605 ) | 0.675 (0.37, 0.94 ) | -2.306 | 0.021* |
|  | Day 7 | 1.3 (0.825 , 1.855 ) | 0.76 (0.47 , 1.48 ) | -2.32 | 0.02* | 1.3 (0.765 , 1.915 ) | 0.575(0.28 , 0.935 ) | -3.325 | 0.001** |
| Eosinophil count (10^9^/ L) | Day 3 | 0.0135 (0 , 0.0525 ) | 0 (0 ,0075 ) | -2.546 | 0.011* | 0.0065 (0 , 0.0425 ) | 0 (0 ,0075 ) | -1.544 | 0.123 |
|  | Day 5 | 0.0095 (0 , 0.04 ) | 0 (0 , 0.01 ) | -2.09 | 0.037* | 0.002 (0 , 0.03 ) | 0 (0 , 0.01 ) | -1.07 | 0.284 |
|  | Day 7 | 0.0645(0.00075,0.1075) | 0.01 (0. , 0.045 ) | -1.83 | 0.067 | 0.025(0.004,0.0915) | 0 (0 , 0.06 ) | -1.902 | 0.057 |
| SAA (mg/L) | Day 3 | 388 (126.25 , 631.25 ) | 626 (266 , 832.25 ) | -1.845 | 0.065 | 472 (138.25 , 716 ) | 571.5 (270.75, 1310.75 ) | -1.09 | 0.276 |
|  | Day 5 | 124 (30 , 284 ) | 416 (183 , 729.75 ) | -3.486 | <0.001** | 221 (71.1 , 402) | 661.5(187.25 , 1017.5 ) | -2.499 | 0.012* |
|  | Day 7 | 21 (10.6 , 91.3 ) | 370 (75.6 , 976.5 ) | -3.973 | <0.001** | 59.55 (18.725 , 207 ) | 432.5 (173.5 , 987.75) | -2.762 | 0.006** |
| CRP(mg/L) | Day 3 | 56.95 (35.225 , 133.5 ) | 91.5 (47.675 , 168.75 ) | -1.291 | 0.197 | 65 (34.2 , 136.75 ) | 103.5 (67.85 , 185.75 ) | -1.885 | 0.059 |
|  | Day 5 | 24.4 (13.6 , 46.4 ) | 54.6 (28.025 , 107.5 ) | -2.344 | 0.019* | 28.8 (17 , 67.6 ) | 81.9 (28.575 , 189 ) | -2.139 | 0.032* |
|  | Day 7 | 9.41 (3.3 , 29.4 ) | 50.3 (9.15 , 97.75 ) | -2.956 | 0.003** | 11.05 (3.3 , 47.97 ) | 82.15 (17 , 140.25 ) | -2.885 | 0.004** |
| Blood urea nitrogen (mg/dl) | Day 3 | 17 (12 , 25.2 ) | 30 (20.75 , 51.25 ) | -3.156 | 0.002** | 20(13.5 , 29.5 ) | 34 (25.5 , 61 ) | -2.465 | 0.014* |
|  | Day 5 | 17.5 (13 , 24.375 ) | 34 (24.15 , 64.25 ) | -3.934 | <0.001** | 21 (14.95 , 29.15 ) | 44 (29.25 , 65.75 ) | -3.591 | <0.001** |
|  | Day 7 | 19.3 (13 , 24 ) | 40.5 (26.55 , 66.75 ) | -4.46 | <0.001** | 22.7 (16 , 31.5 ) | 48.8 (38.25 , 78 ) | -3.736 | <0.001** |
| Creatinine(mg/dl) | Day 3 | 0.77 (0.6525, 1.0825) | 1.36 (0.965 , 1.9625 ) | -2.897 | 0.004** | 0.89 (0.6375, 1.245) | 1.475 (1.0525 , 2.3175) | -2.631 | 0.009** |
|  | Day 5 | 0.795 (0.66, 1.005 ) | 1.19 (0.85 , 2.03 ) | -3.644 | <0.001** | 0.85 (0.75, 1.135 ) | 1.19 (1.07 , 2.57) | -3.029 | 0.002** |
|  | Day 7 | 0.77 (0.5975 , 0.99 ) | 1.305 (0.95 , 2.0625 ) | -3.796 | <0.001** | 0.905 (0.7325 , 1.18 ) | 1.59 (1.07 , 2.43 ) | -3.15 | 0.002** |
| Uric acid (mg/dL) | Day 3 | 4.45 (3.825 , 6.325 ) | 6.55 (4.725 , 8.3 ) | -2.197 | 0.028* | 4.7 (3.875 , 6.61 ) | 7.2 (5.4 , 8.425 ) | -2.208 | 0.027* |
|  | Day 5 | 4.2 (3.875 , 6.55 ) | 6.5 (4.325, 8.25 ) | -1.802 | 0.072 | 4.65 (3.675 , 7.0475 ) | 7.1 (4.875, 8.925 ) | -1.943 | 0.052 |
|  | Day 7 | 5 (3.55 , 5.6 ) | 5.5 (3.9 , 7.8 ) | -1.136 | 0.256 | 4.63 (3.425 , 5.975) | 6.45 (4.2125 , 8.1 ) | -1.997 | 0.046* |
| Creatine kinase (U/L | Day 3 | 67 (41.5 , 127.75 ) | 253 (96.75 , 340.75 ) | -2.831 | 0.018* | 95 (50 , 268.5 ) | 304 (142.5 , 4099 ) | -1.789 | 0.074 |
|  | Day 5 | 65.5 (38.25 , 88 ) | 198.5 (92.75 , 715.25 ) | -2.807 | 0.005** | 69.9 (41 , 158 ) | 255 (218 , 1117.5 ) | -3.197 | 0.001** |
|  | Day 7 | 44 (31.75 , 64.25 ) | 128.5 (60.25 , 484.75 ) | -2.222 | 0.026* | 52 (33.75 , 128.25 ) | 289.5 (114.5 , 833 ) | -2.861 | 0.004** |
| LDH (U/L) | Day 3 | 373 (352.5 , 498.25 ) | 614 (424.25 , 881.5 ) | -2.671 | 0.008** | 432 (370.75 , 750 ) | 644 (383.25 , 933.75 ) | -0.952 | 0.341 |
|  | Day 5 | 398 (329.5, 494 ) | 602.5 (473.75 , 840.5 ) | -2.86 | 0.004** | 519 (397.75, 750 ) | 537 (452 , 841 ) | -0.424 | 0.672 |
|  | Day 7 | 329 (272.5 , 576 ) | 534 (485 , 735.5 ) | -1.653 | 0.098 | 513 (280.5 , 576 ) | 542 (491 , 738) | -1.652 | 0.099 |

SAA, Serum amyloid A; CRP, C-reactive protein; CK-MB, Creatine kinase-MB; LDH ,lactate dehydrogenase ;*p< 0.05 and **p< 0.01.

Data are expressed as mean ±SD, median (25 to75% percentile), or number (%). P value ˂0.05 is significant

Supplementary Table 2: Comparison of initial SAA levels between patients with and without comorbidities across different groups

| Patients’ groups | Medium SAA level in patients without comorbidities | Medium SAA level in patients without comorbidities | Z | P | SIG |
| --- | --- | --- | --- | --- | --- |
| Moderate/severe group | 538 (371.5-826) | 129 (66.2-903) | -1.633 | 0.102 | NS |
| Critical severe group | 406 (284-753) | 455(150-1070) | -0.452 | 0.651 | NS |
| Survival group | 496.5 (320-762.5) | 350 (72.4-1028) | -0.409 | 0.682 | NS |
| Non-survival group | 550 (347-753) | 374.5(170.3-970.3) | -1.934 | 0.053 | NS |

Supplementary Table 3 : Dynamic changes of SAA and CRP in COVID‑19 patients.

| delta change | Moderate /severe | Critically ill | Z | p | survivor | Non survivor | Z/t | p |
| --- | --- | --- | --- | --- | --- | --- | --- | --- |
| SAA. between day 3& day1 | -0.10898(- .424,0.476) | 0.14315(-0.295,0.884) | -0.792 | 0.428 | -0.128(-0.49,0.52) | 0.4131(-0.142,0.89) | -1.431 | 0.152 |
| SAA. between day 5& day 1 | -0.73902(-0.856,0.176) | -0.08481(-0.528,2.017) | -2.298 | 0.022* | -0.534(-0.81,0.54) | -0.0883 (-0.506,2.033) | -1.593 | 0.111 |
| SAA between day 7& day 1 | -0.85795(-0.971,-0.649) | -0.1271(-0.903,0.389) | -2.372 | 0.018* | -0.765(-0.96,-0.14) | 0.1833 (-0.672,2.037) | -2.286 | 0.022* |
| SAA between day 5& day 3 | -0.5625(-0.76,-0.183) | -0.26468(-0.53,0.48) | -2.627 | 0.009** | -0.418(-.65,0.12) | -0.3847 (-0.559,1.973) | -1.046 | 0.295 |
| SAA between day7& day 3 | -0.86547(-0.968,-0.628) | -0.36752(-0.892,0.391) | -2.814 | 0.005** | -0.731(-0.95,-0.38) | -0.0323(-0.772,0.625) | -2.357 | 0.018* |
| SAA between day7& day 5 | -0.60368(-0.883,-0.459) | -0.51268(-0.795,0.464) | -1.275 | 0.202 | -0.605(-0.81,-0.32) | -0.136 (-0.749,0.9713) | -1.619 | 0.105 |
| CRP between day 3& day 1 | 0.21909±1.815712 | 0.41917± 2.419805 | -0.332 | 0.741 | 0.366 ± 2.4275 | 0.18467 ± 0.630152 | 0.256 | 0.799 |
| CRP between day 5& day 1 | -0.21455±1.3468 | 0.10914± 1.444774 | -0.811 | 0.421 | -0.170 ± 1.3216 | 0.3504 ± 1.5965 | -1.127 | 0.265 |
| CRP between day 7& day1 | -0.1334± 3.1530 | 0.06079± 1.634918 | -0.264 | 0.793 | -0.259 ± 2.541 | 0.64946 ± 2.126586 | -1.113 | 0.271 |
| CRP between day 5& day 3 | -0.39449 ±0.512906 | -0.0709± 0.939278 | -1.519 | 0.137 | -0.308± 0.6227 | 0.04012± 1.130563 | -1.357 | 0.181 |
| CRP between day 7& day 3 | -0.57542±0.748939 | 0.1434± 1.55784 | -2.063 | 0.047* | -0.376± 1.0213 | 0.3257± 1.804729 | -1.68 | 0.1 |
| CRP between day 7& day 5 | -0.38604±0.645039 | 0.1815± 1.1603 | -2.116 | 0.041* | -0.275± 0.7918 | 0.46352± 1.295739 | -2.364 | 0.022* |

Abbreviations: SAA, Serum amyloid A ; CRP, C-reactive protein;*p< 0.05 and **p< 0.01.
